# Histomorphic Analysis of UV-C Radiation on Osseointegration of Titanium Implants in the Rabbits

**DOI:** 10.30476/DENTJODS.2021.91574.1592

**Published:** 2022-12

**Authors:** Reza Vafadoost, Mohammad Reza Shabahangfar, Ahad Khoshzaban, Hamid Ahmadian Moghadam

**Affiliations:** 1 Researcher, Dept. of Periodontics, College of Dentistry, Islamic Azad University of Tehran Medical Sciences, Tehran, Iran; 2 Dept. of Periodontics, College of Dentistry, Islamic Azad University of Tehran Medical Sciences, Tehran, Iran; 3 Researcher, Dept. of Genetics, Iranian Institute for Reduction of High-Risk Behaviors, Tehran University of Medical Sciences, Tehran, Iran

**Keywords:** Osseointegration, Ultraviolet Rays, Dental Implants, Animal Model

## Abstract

**Statement of the Problem:**

Unsuccessful implant integration leads to pain and implant mobility. Implant photo-functionalization by ultraviolet (UV) light has been suggested as a method that may stimulate osseointegration.

**Purpose:**

This study was conducted to analyze the histopathological feature of the titanium implant surface upon treatment with UV-C wave.

**Materials and Method:**

In this interventional study, twenty rabbits were enrolled. In the treatment groups, the titanium implants, irradiated earlier with UV-C for four hours laterally, were inserted in one of the femur bones. In the control group, the titanium implants without irradiation were inserted in the other femur bone of the rabbits. After two and four weeks, the animals were sacrificed, and then the samples were histologically and histo-morphometrically analyzed. In addition, the amounts of new bone formation, bleeding, and inflammation were recorded, and the data were subjected to statistical analysis.

**Results:**

The results confirmed that UV-C irradiation to titanium implants significantly improved new bone formation (p< 0.001). However, no significant new bone formation was observed between two and four weeks after implant insertion (p< 0.098).

**Conclusion:**

The study results showed that irradiating titanium implants with UV-C for four hours significantly improves osseointegration and new bone formation but does not considerably affect inflammation or bleeding around the implant. The study suggests that UV-C radiation can increase the success rate of implant treatment.

## Introduction

The dental implant has become a routine clinical practice to replace a missing tooth. It helps patients feel more comfortable and functional than conventional prostheses [ 1 - 3 ]. Osseointegration is a practical and direct connection between the implant and live bone required for successful implant integration [ 2 , 4 ]. Unsuccessful implant integration leads to pain, implant mobility, and mastication force impairment [ 5 - 6 ]. Techniques such as sandblasting are widely used to increase surface roughness and improve osseointegration [ 7 ]. 

However, roughed surfaces are strongly associated with plaque accumulation [ 8 ]. Meanwhile, implant photo-functionalization by UV light has been suggested as an effective method to stimulate osseointegration [ 9 - 10 ]. The UV photo-functionalization was discovered in 1977 and defined as a change in titanium surface upon UV treatment. The treatment changes the hydrophobic features of the titanium surface into super-hydrophilic and enhances its biological capabilities [ 9 - 12 ]. UV photo functionalization is a phenomenon of surface modification by exposure to ultraviolet rays that alters the surface's physicochemical features and improves its biological capabilities [ 13 ]. Photo functionalization of titanium implants by UV is discovered as a simple and effective tool for osseointegration [ 10 , 14 - 15 ]. This discovery shows that the physicochemical property of the titanium surface changes and transforms the hydrophobic property of the titanium surface into a solid hydrophilic feature. The phenomena are practically applied in microbiology and improve several biological capabilities [ 11 - 12 , 16 ]. 

Moreover, evidence suggests that photo-functionalization may improve osseointegration in the initial healing period [ 17 ]. Furthermore, the evidence indicates that photofunctionalization by UV may improve attachments, retention, and a functional cascade of osteogenic cells [ 13 ]. It is noteworthy that UV photo-functionalization is novel, simple, and low cost. However, further studies should validate these findings [ 13 ]. The potential of surface modification to make a successful dental implant material is highly associated with success in vitro and clinical studies. Therefore, a deep understanding of tissue response and osseointegration by dental implants is required [ 18 ]. Hence, this study was conducted to analyze the histopathological feature of the titanium implant surface upon treatment with UV-C wave. 

## Materials and Method

### UV light treatment

The titanium implants (3.25mm×8.5mm fixtures, Charum Medimecca, Korea) were irradiated by a specially manufactured UV-C light generator using 15W bactericidal lamps (Philipps, Netherlands) for at least four hours. The intensity of light was 5mW.cm-2 (λ=253±7)nm. 

### Animals

**Figure 1:**
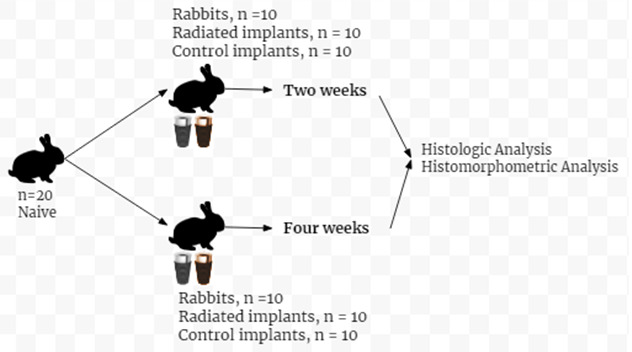
The figure illustrates the study protocol. A total of 20 naïve rabbits, 20 radiated implants, and 20 control implants have been used in this study

The study protocol is illustrated in Figure 1. The experiment was conducted according to the guideline care of laboratory animals by the Tehran University of Medical Sciences. The study complied with the ARRIVE guidelines and National institutional guidelines for the care and use of laboratory animals. In this study, twenty healthy Albino rabbits were enrolled. The animals were obtained from the Pasture Institute (Iran). The animals were housed in separate cages under standard temperature, humidity, and regular daylight cycle for one week. The animals weighed 2-3 kg and were approximately the same age. The animals received an everyday nutritional regime and typically gained weight. In addition, a veterinarian ensured the systemic health of rabbits. Through the surgery process, two implants were placed in the femur bone of each animal. One implant was treated by UV-C light; the other was not treated and was considered a control implant. The animals were divided into two groups and housed for two and four weeks. The animals were sacrificed at the end of the treatment period and the samples were subjected to microscopic analysis.

### Surgery

**Figure 2:**
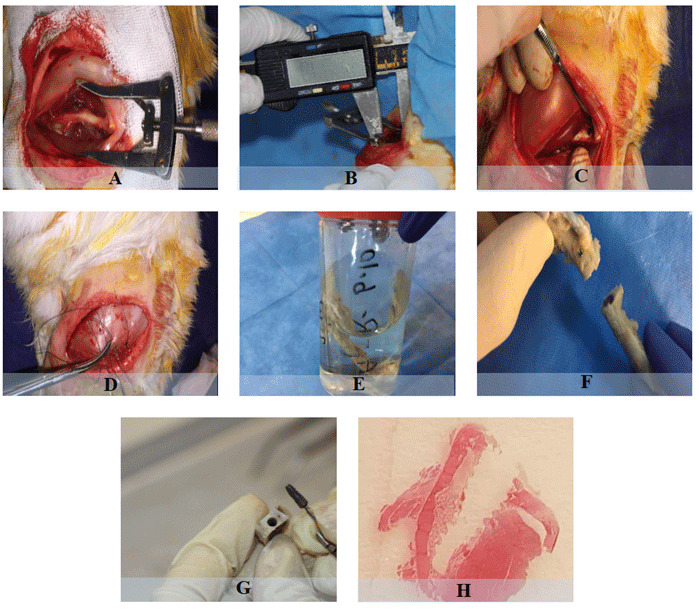
Sample staining and micro-cutting

**Figure 3:**
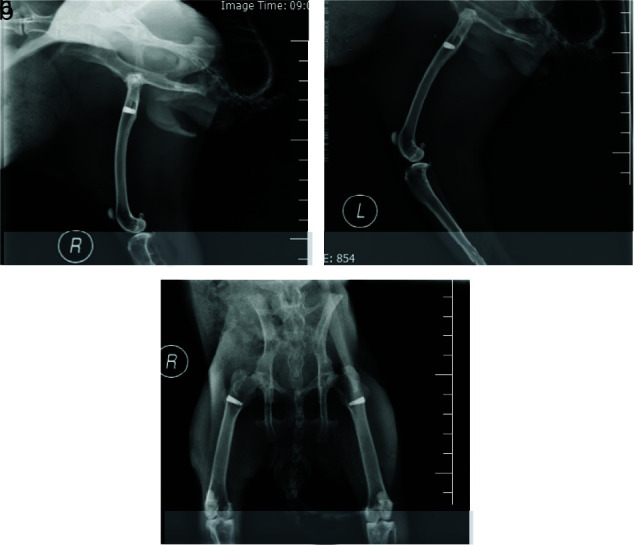
Front view of the implant in the femur bone

The surgery was performed according to the protocol formerly provided by Gehrke *et al.* [ 19 ]. Furthermore, the experimental protocol was conducted according to the role and guidance provided by the Tehran University of Medical Science. For this purpose, twenty rabbits were obtained. The animals were slightly transferred into the surgery room. The operating table was disinfected with ethanol (70%) solution. Then, the animals were anesthetized with Xylazine (2%, 7mg.kg-1) and Ketamine hydrochloride (10%, 44mg.kg-1) intramuscularly injected into the superior-lateral quarter of the quadriceps muscle. The skin was shaved, and the area of the proximal femur bone was washed with Betadine solution. The Prilocaine-Flypressin 1% was subcutaneously administered at the surgery site to improve anesthesia and control bleeding. An incision was made to expose the bone of both proximal femurs. A cavity in the bone was made with burs under saline irrigation. The implant from each respective group was inserted into the cavity of each femur bone. The implant was positioned with the marginal border of the femur bone under controlled torque (20 N). The incision was sutured, and a dose of Benzetacil (600,000 IU) was used. After surgery, the animal was housed in separate cages with a controlled atmosphere (21○C), 12 hours light cycle and a diet generally used according to the veteran guideline. No death occurred after the post-operation period. Finally, the animals were sacrificed by intravenous overdose of 2ml ketamine and 1ml Xylazine at the end of the experiment. The femur bone was removed and placed in a formalin (10%) solution. Finally, the samples were microscopically analyzed (Figures 1 and Figure 2). The correct replacement of implants was confirmed by radiographic examination (Figure 3).

### Microscopic analysis

#### Histologic Analysis

The samples were dehydrated by ethanol, and for decalcification, the samples were embedded in acid formic (10%, 21C) for 21 days, followed by washing for 12 hours. The un-decalcified cut contained the central part of each implant (15mm) using a grinding system. The samples were stored in formalin (10%) for a week. Later the samples were stained with Hematoxylin and Eosin for histologic analysis. 

A pathologist evaluated the samples, while the groups were blinded to the pathologist. The samples were assessed on a digital image (E450; Nikon, Japan) taken at 40× magnification using Iranian histomorphometric analysis software (IHMA v1, Shaheed Beheshti University, Iran). The presence of newly formed bone, amount of bleeding, and tissue around the implant, including bone, and fibrous tissues, were evaluated in the first group after two weeks and in the second group after four weeks upon sacrificing animals (Figure 1).

### Histo-morphometric Analysis

**Figure 4:**
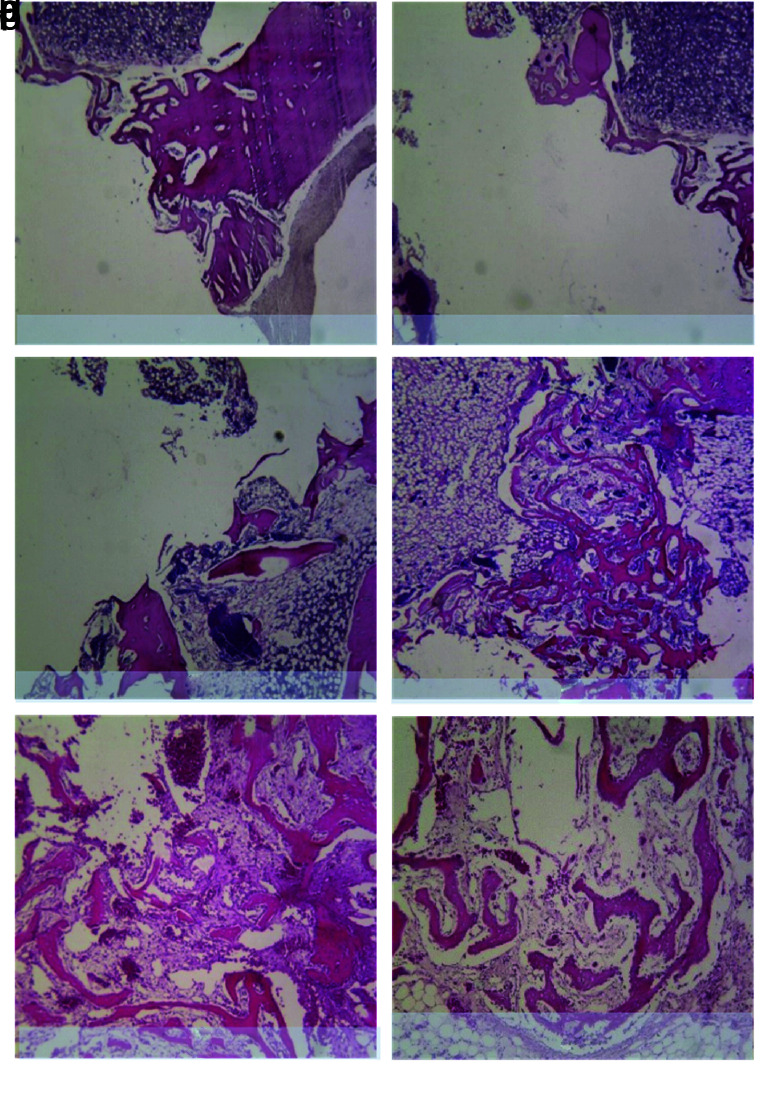
New bone formation in the control group with 40x magnification

For the histo-morphometric analysis, the prevalence of the newly formed bone was recorded. It calculated the specimens at the largest diameter of defects on a digital image (E450; Nikon, Japan) taken at 40× magnification using Iranian histo-morphometric analysis software (IHMA v1, Shaheed Beheshti University, Iran). The percent of cortical one formation and bone marrow formation was recorded (Figure 4). 

### Statistical Analysis

The sample size was calculated according to the previous study by Gehrke *et al.* [ 19 ] and below the mathematical model. The sample size for the current study was calculated as 20 rabbits. The normality test was used to reduce the chance of false-positive results using the Kolmogorov-Smirnov test. The recorded data were subjected to a two-way analysis of variance (ANOVA), followed by Tukey's post hoc mean comparison test. Non-parametric values were analyzed using the chi-square test. All statistical studies were conducted using SPSS 21 software. The differences with *p* Values lower than 0.05 are considered significant.

$n=\frac{\delta d^{2}{\left(\mathrm{z\alpha}+\mathrm{z\beta}\right)}^{2}}{{\left({µ}_{1}-{µ}_{2}\right)}^{2}}$

δ_d_≃6.73,α=0.01,β=0.05,µ_1_-µ_2_=6.39

n: least required sample, µ1: an average of data in the first group, which was obtained from previous studies, µ1: average of data in the second group, which was obtained from previous studies, α: the probability of type one error that shows a significant level of the study, β: the probability of type 2 error or statistical power of the study. (µ1-µ2): the difference between the averages of values in both groups that expressed the significant difference between the two groups. 

## Results

The results of the normality test using the Kolmogorov-Smirnov test confirmed normal distribution between control and treatment groups in all time trials. Moreover, results of chi-square analysis showed that there was no significant difference between inflammation (p< 0.487), bleeding (p=1.0), peri-implant tissues (p=0.086). In other words, exposure to UV-C had no significant effect on inflammation, bleeding, and tissues forming around the implant. However, results showed that irradiation of the titanium implants significantly affected the cancellous and cortical bones (p< 0.05).

### Effect of UV-C radiation of implants on cortical bone

**Figure 5:**
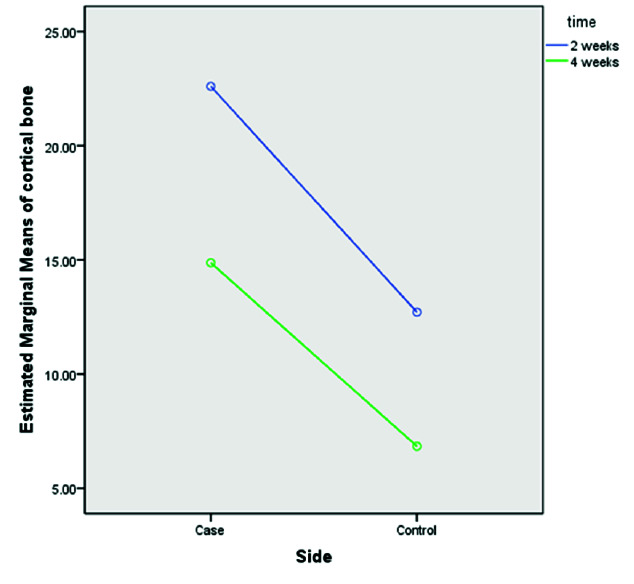
The diagram shows the interaction between the mean of cortical bone formation in treatment and control groups with different time trials

ANOVA test results showed no significant difference for cortical bone formation in different time trials (p= 0.074, Table 1). However, the chi-square test showed that the percentage of cortical bone formation was significantly higher in the treatment group (p=0.001). Moreover, results showed that the interaction between groups and time was not significantly different (Figure 5).

**Table 1:** Result of ANOVA analysis for the percent of cortical bone

	Time	Mean	Std. Deviation	Min	Max	*p* Value
Treatment	Two weeks	34.77	6.04	8.15	66.08	0.074
	Four weeks	25.86	6.57	0.0	58.84	
Control	Two weeks	19.78	3.23	8.0	37.20	
	Four weeks	8.95	2.14	0.0	21.55	

### Effect of UV-C radiation of implants on cancellous bone

**Figure 6:**
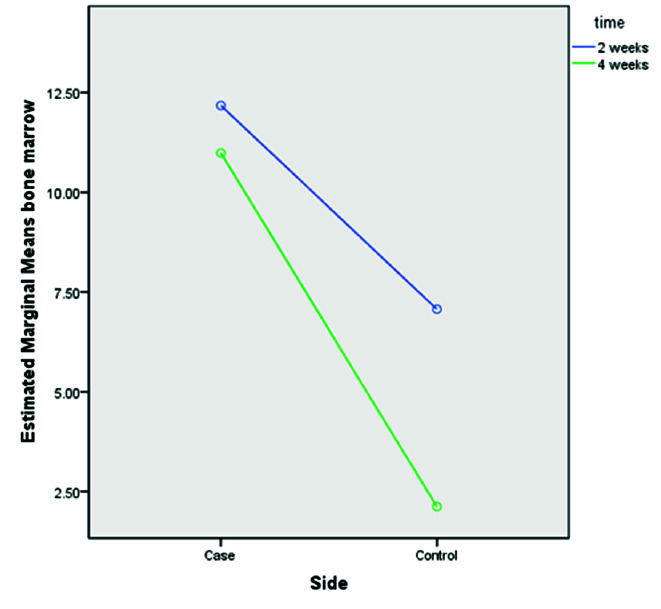
The diagram shows the interaction between the mean of cortical bone formation in treatment and control groups with different time trials

ANOVA test results showed no significant difference for cancellous bone formation in different time trials (p= 0.231). The chi-square test showed that the percent of cancellous bone formation in two weeks was higher than in four weeks. However, this difference was not statistically significant (p= 0.701, Table 2). Moreover, results showed that UV-C radiation significantly improved cancellous bone formation by four weeks (p= 0.035). It is noteworthy that cancellous bone formation between two weeks (p= 0.014) and four weeks (p= 0.045) was significantly higher than in the control group. Moreover, results showed that the interaction between groups and time was not significantly different (p= 0.112, Figure 6).

**Table 2:** Result of ANOVA analysis for the percent of cancellous bone

	Time	Mean	Std. Deviation	Min	Max	*p* Value
Treatment	Two weeks	12.17	2.96	0.0	27.60	0.231
	Four weeks	10.98	3.11	0.0	31.7	
Control	Two weeks	7.06	1.72	0.0	19.0	
	Four weeks	2.12	0.95	0.0	8.48	

### Effect of UV-C radiation of implants on new bone formation

**Figure 7:**
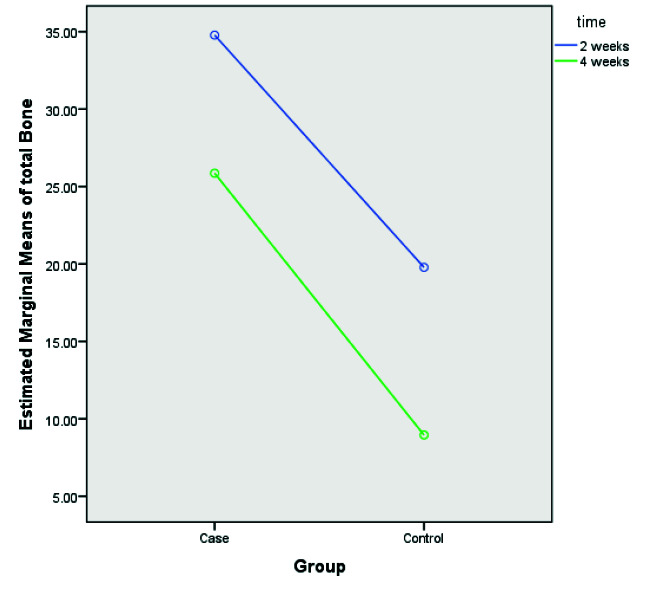
The diagram shows the mean of total bone formation in treatment and control groups with different time trials

ANOVA test results showed no significant difference for total new bone formation in different time trials (p= 0.098). The chi-square test showed that although the percentage of cancellous bone formation in two weeks was higher than in four weeks, this difference was not statistically significant (p= 0.811, Table 3). However, results showed that total new bone formation was significantly higher than the control group (p= 0.001). Moreover, results showed that the interaction between groups and time was not significantly different (Figure 7).

**Table 3:** Result of ANOVA analysis for the percent of new bone formation

	Time	Mean	Std. Deviation	Min	Max	*p* value
Treatment	Two weeks	34.77	6.04	8.15	66.08	0.098
	Four weeks	25.86	6.57	0.0	58.84	
Control	Two weeks	19.78	3.23	8.0	37.20	
	Four weeks	8.95	2.14	0.0	21.55	

## Discussion

In this study, the histo-morphometric effect of UV-C irradiation on osseointegration of dental implants was evaluated. Results showed that new bone formation on the implant exposed to UV-C was significantly higher than in control groups. An increase in bone formation is affected by several factors, including (1) an increase in protein absorption, (2) removal of hydrocarbons from titanium surface, (3) increase in osseo-conductive on titanium surface [ 10 ], (4) removal of biologic pollutants [ 20 ], (5) increase in activity of alkaline phosphatase, (6) calcium mineralization [ 21 ], (7) improvement of hydrophilic activity of titanium surface [ 21 ], (8) increase in osteoblast migration, (9) increase in osteoblast attachment, increase in growth and development of osteoblasts [ 10 ], (10) increase in cellular distribution, and (11) improve in cytoskeletal structure and Vinculin expression [ 22 ]. It is noteworthy that the processes mentioned above are not separate phenomena; for instance, an increase in protein attachment increases the attachment of osteoblasts, and the growth of osteoblasts leads to cellular differentiation. These phenomena consequently lead to osteogenesis and osseointegration [ 10 ]. Evidence shows that the contamination of the titanium surface by saliva impaired the osteoblastic function of the titanium surface [ 20 ]. In other words, UV radiation clears biological pollutants and consequently changes the osteoblastic function of the titanium surface [ 20 ]. Furthermore, much evidence revealed that UV-C has antimicrobial potential [ 23 - 25 ]. The results of our study also suggest that UV-C could improve osseointegration. 

Another evidence shows that UV radiation improves osteoblastic functions, such as alkaline phosphatase activity and calcium mineralization [ 21 ]. It was revealed that UV radiation changes the physicochemical properties of the titanium surface and significantly improves osteoblast attachment and function [ 21 ]. Moreover, results showed that a robust hydrophilic feature appears upon UV irradiation on the titanium surface [ 21 ]. The future reduces the water-droplet angle with the titanium surface to less than five degrees. Notably, the non-irradiated titanium surface is a hydrophobic and water-droplet angle with the titanium surface to less than five degrees [ 21 ]. In this background, the results of our study suggest that improvement in osseo-integration would be due to the inducement of a hydrophilic feature on the titanium surface that increases initial osteoblast attachment to the titanium surface. 

Furthermore, our study found that new bone formation significantly increased by UV-C irradiation on titanium dental implants after two and four weeks. However, in both groups, no significant difference was observed in new bone formation between two and four weeks of the experiment. Similar evidence revealed that exposure to titanium implants with UV radiation for four weeks significantly increased bone regeneration [ 26 ]. However, the extension of that experiment to twelve weeks showed no significant difference in osseo-integration in the treatment groups with the control group [ 26 ]. It is noteworthy that the impacts of UV irradiation during long-term treatment are affected by the type of cell, experiment length, intensity, UV wavelength, and implant surface texture [ 27 - 28 ]. Improved osseo-integration on UV irradiated implant for extending four months is due to increased mineralization between bone and implant, elevation in Aluminum concentration, and increased oxygen concentration on implant surface [ 26 - 28 ]. Another study revealed that exposure to a titanium implant for twelve minutes after twelve to twenty-four days significantly increased bone regeneration and osseointegration in rats [ 29 ]. 

Our study observed no significant bone regeneration between two and four weeks of treatment. It could be due to two reasons: (1) the physiology of bone regeneration in rabbits changes after two weeks. In which bone formation reduces and enters the remodeling phase [ 27- 29 ], (2) the studied animal has a low amount of cortical bone, and the bone primarily contains bone marrow that resists new bone formation [ 30 ]. Furthermore, inflammation, bleeding, and fibrosis issues were evaluated in our study. However, the non-parametric method's histologic analysis revealed no significant difference between the control and treatment groups. That suggests UV radiation had no significant effect on inflammation and bleeding around the implant. 

## Conclusion

The study showed that a titanium implant irradiated with UV-C for four hours significantly improved osseointegration and new bone formation. However, prolonging the experiment from two to four weeks had no significant effect on bone formation. Therefore, the study suggests that UV-C radiation can increase the success rate of implant treatment. 

## Acknowledgement

The authors appreciate the Journal of Dentistry for peer review and publishing the manuscript. All experiment procedures were conducted according to rules of experimental-animal ethics at Azad University of medical science, Medical Branch. The authors report no relevant financial conflicts. The authors acknowledge receiving no funds from any institute. RV conducted the experiment and wrote the manuscript; MS and AK equally have read and commented on the manuscript. HA analyzed, read, and edited the manuscript.

## Conflict of Interest

The authors acknowledge that there is no competing interest.
